# The utilization of primary healthcare services among frail older adults – findings from the Helsinki Birth Cohort Study

**DOI:** 10.1186/s12877-022-02767-4

**Published:** 2022-01-26

**Authors:** Jenni N Ikonen, Johan G Eriksson, Mikaela B von Bonsdorff, Eero Kajantie, Otso Arponen, Markus J Haapanen

**Affiliations:** 1 Department of General Practice and Primary Health Care, University of Helsinki, Helsinki, Finland; 2 Folkhälsan Research Center, Helsinki, Finland; 3 Yong Loo Lin School of Medicine, Department of Obstetrics and Gynecology and Human Potential Translational Research Programme, National University of Singapore, Singapore, Singapore; 4 Singapore Institute for Clinical Sciences (SICS), Agency for Science, Technology and Research (A*STAR), Singapore, Singapore; 5 Gerontology Research Center and Faculty of Sport and Health Sciences, University of Jyväskylä, Jyväskylä, Finland; 6 Population Health Unit, Finnish Institute for Health and Welfare, Helsinki, Finland; 7 PEDEGO Research Unit, MRC Oulu, Oulu University Hospital, University of Oulu, Oulu, Finland; 8 Department of Clinical and Molecular Medicine, Norwegian University of Science and Technology, Trondheim, Norway; 9 Children’s hospital, Helsinki University Hospital, University of Helsinki, Helsinki, Finland; 10 Department of Radiology, Tampere University Hospital, Tampere, Finland; 11 Faculty of Medicine and Health Technology, Tampere University, Tampere, Finland; 12 Department of Medical Epidemiology and Biostatistics, Karolinska Institute, Stockholm, Sweden

**Keywords:** Frailty, Primary healthcare, Physiotherapy, General practice, Remote contact

## Abstract

**Background:**

The impact of frailty on primary healthcare service use, especially general practice office visits and remote contacts, is currently unknown. Further, little is known about the association of frailty with physiotherapy contacts.

**Methods:**

We examined the utilization of primary healthcare services among 1064 participants from the Helsinki Birth Cohort Study between the years 2013 and 2017. Frailty was assessed based on Fried’s frailty criteria at mean age of 71.0 (2.7 SD) years in clinical examinations between the years 2011 and 2013. General practice office visits and remote contacts, the total number of general practice contacts, physiotherapy contacts, and the total number of primary healthcare contacts were extracted from a national Finnish register. We analyzed the data with negative binomial regression models.

**Results:**

Of the 1064 participants, 37 were frail (3.5%) and 427 pre-frail (40.1%); 600 non-frail (56.4%) served as a reference group. Frailty was associated with general practice office visits (IRR 1.31, 95% CI=1.01-1.69), physiotherapy contacts (IRR 2.97, 95% CI=1.49-5.91) and the total number of primary healthcare contacts (IRR 1.41, 95% CI=1.07-1.85). Pre-frailty predicted the use of general practice remote contacts (IRR 1.39, 95% CI=1.22-1.57) and the total number of general practice contacts (IRR 1.25, 95% CI=1.12-1.40).

**Conclusions:**

Frailty increases the overall primary healthcare service use whereas pre-frailty is associated with the use of general practice services, especially remote contacts. Primary healthcare needs measures to adapt healthcare services based on the needs of rapidly increasing number of pre-frail and frail older adults and should consider preventative interventions against frailty.

## Introduction

During the past two decades there has been an emerging concern regarding whether healthcare systems can meet the requirements of the rising number of older adults. As an increasing demographic group, older adults with ageing-related chronic conditions utilize healthcare services more than younger populations on average [1]. Primary healthcare, the frontline of healthcare systems in most countries, provides crucial disease prevention, diagnostics, and long-term care to older adults [2]. This is particularly true in Nordic countries, like Finland, where the private healthcare sector is relatively small and supplements universal public healthcare [3]. To better suit primary healthcare services for the increasing demands of older adults due to population ageing, it is important to find ways to prioritize preventive interventions and identify factors that lead to frequent healthcare use.

Individual diversity in health at older age, however, is large [4]. The concept of frailty, representing a geriatric condition characterized by exceptional vulnerability to stressors [5, 6], might be a promising approach to understand the health differences and different primary healthcare utilization rates among older adults. While frailty is associated with adverse health outcomes including falls, delirium, and mortality, it has also been associated with increased utilization of healthcare services [5, 6]. Therefore, it might be a promising way to detect the frequent healthcare users among older adults for planning targeted services and preventative measures. Moreover, the prevalence of frailty is expected to increase along with population ageing [6] creating a need to understand the primary healthcare utilization patterns among frail patients. Literature regarding frailty and the use of primary healthcare services is, however, mainly based on studies about the association between frailty and general practitioner visits [7–14] with little focus on the total number of primary healthcare contacts. No studies have separated general practice contacts into office visits and remote contacts to reveal the possible demand for less expensive remote contacts among frail older adults. Furthermore, few studies have examined the association of frailty with the utilization of physiotherapy services, the first-line treatment of the functional decline of frail older adults [11, 15].

The aim of the present study is to investigate the possible association of frailty with the utilization of primary healthcare services including general practice office visits and remote contacts, physiotherapy, and the total utilization of general practice contacts and primary healthcare contacts. We hypothesized that frailty would be associated with the use of all categories of primary healthcare services.

## Methods

### Study population

**Fig. 1:**
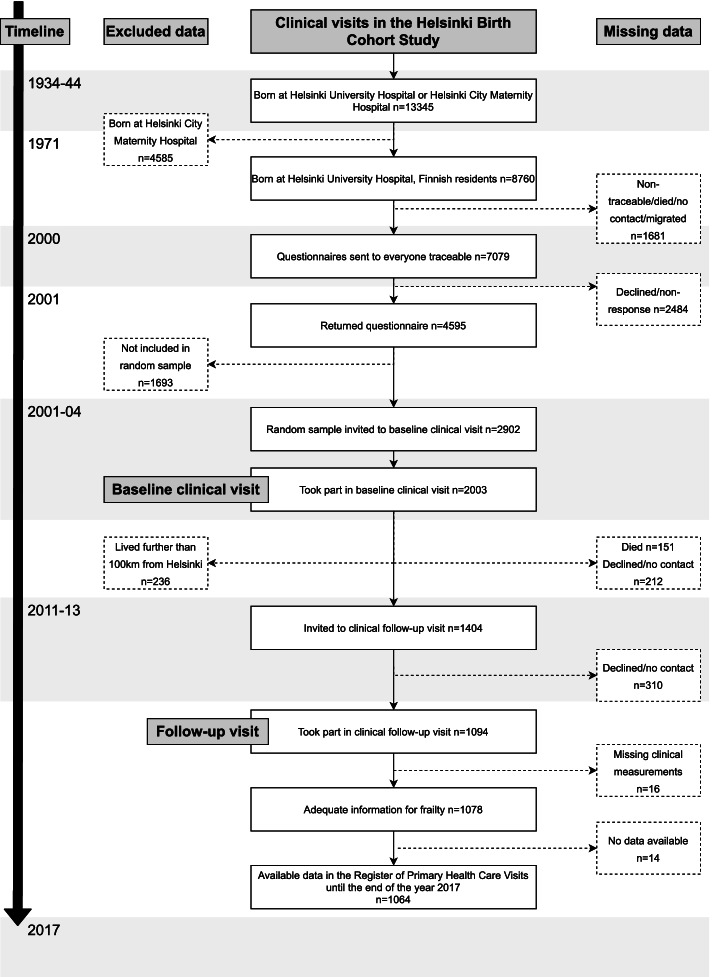
A flowchart of the selection of the study participants

The present study population is a sub-population of the Helsinki Birth Cohort Study of 8760 individuals who were born at Helsinki University Hospital between the years 1934 and 1944. A randomly selected sub-sample of 2003 individuals participated in clinical examinations between the years 2001 and 2004. As seen in the flowchart in Fig. 1, by the clinical re-examinations performed between the years 2011 and 2013, 151 individuals had died, 212 declined to participate in the follow-up study and 236 lived further than 100 km from Helsinki. Of the remaining contacted 1404 individuals, 1094 participated in the re-examination between the years 2011 and 2013 [16]. Of these, 1078 had adequate information for assessing frailty. Of these, 1064 had consistent data in the nationwide Register of Primary Health Care Visits (AvoHilmo) between the years 2013 and 2017 forming the study population. All individuals provided written informed consent before involvement in any clinical procedures. The clinical study was approved by the Coordinating Ethics Committee of the Hospital District of Helsinki and Uusimaa.

### Frailty classification

Frailty was assessed with the five criteria of Fried’s frailty phenotype [5] in the clinical examinations between the years 2011 and 2013 as previously described [17–19]. The criteria included weight loss, exhaustion, low physical activity, weakness, and slowness. Briefly, questionnaires were used to inquire about a recent weight loss of at least 5 kg, exhaustion in 3 or more days a week and total physical activity of less than 1 h a week. Grip strength was measured and belonging to the lowest quintile of participants according to sex and the body mass index (BMI) met the criterion of weakness. The criterion of slowness was assessed by measuring maximal walking speed stratified by sex and height and met among those who belonged to the lowest quintile. Participants were classified as frail if they met three or more criteria, pre-frail if one or two criteria and non-frail if no criteria were met.

### Primary healthcare utilization data

**Fig. 2:**
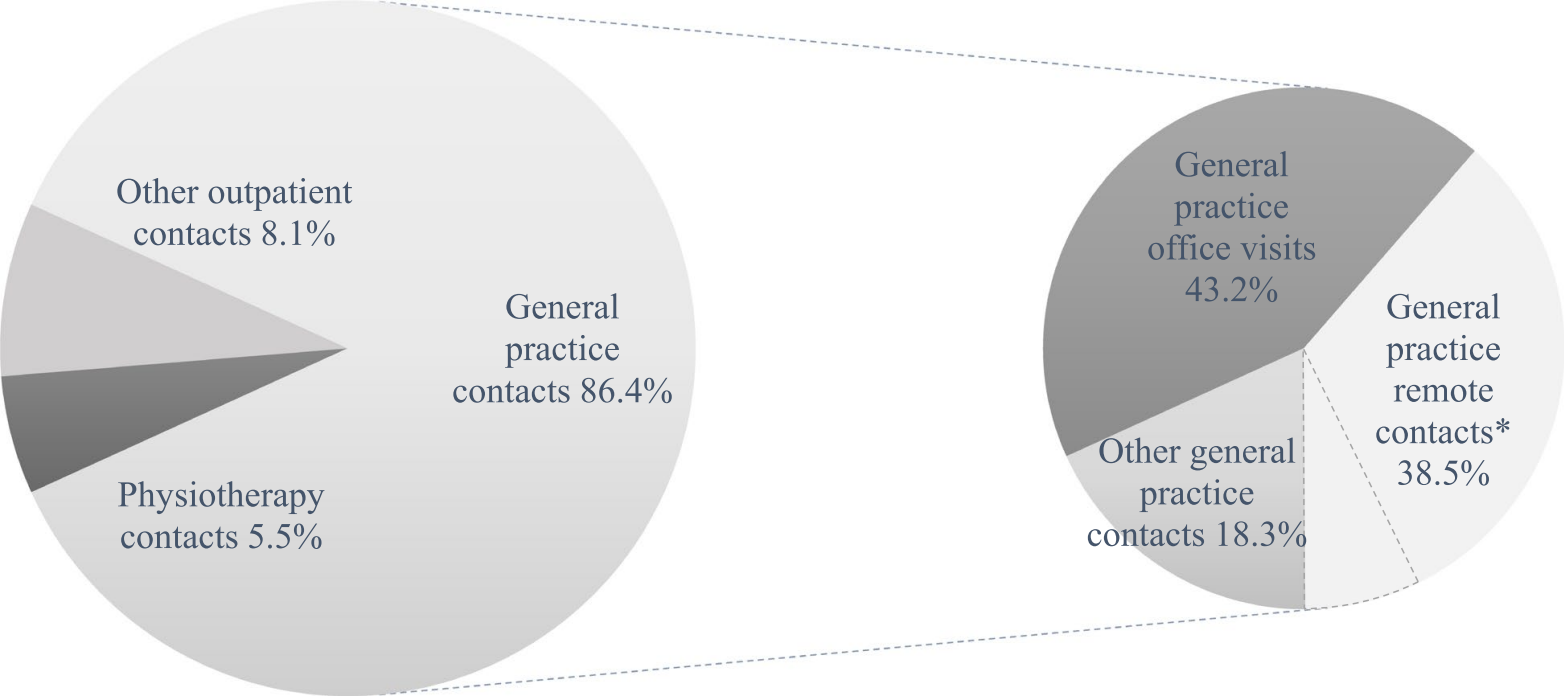
The distribution of all primary healthcare visits during the follow-up. The distribution of all primary healthcare contacts is presented on the left and, on the right, the fractionated sub-group of general practice contacts. *General practice remote contacts consist of phone calls (31.3%) and letters (7.2%); the two proportions are separated with a dash line in the figure

Data on primary healthcare utilization were obtained from the Register of Primary Health Care Visits (AvoHilmo) [20], a national register maintained by the Finnish Institute of Health and Welfare, which encompasses all primary healthcare contacts registered in the public healthcare centers in Finland. The data retrieval started at the beginning of the year 2013, after finishing frailty classification among the study participants, and finished at the end of the year 2017 spanning 5 years of follow-up. Overall, we were able to retrieve more than 60 000 outpatient primary healthcare contacts: 86.4% being general practice, 5.5% physiotherapy, and 8.1% other outpatient contacts including, for example, podiatry, medical certificates, and occupational therapy. As shown in Fig. 2, general practice contacts were further divided into three categories including general practice office visits, general practice remote contacts including both letters and phone calls as means of communication, and other general practice contacts. To further clarify, general practice visits included both general practitioner visits and general practice nurse visits. Other general practice contacts, in turn, consisted of consultations, document notations, and other medical work.

### Outcome variables

General practice office visits and general practice remote contacts were included in the regression analyses while other general practice contacts were excluded since the category contained heterogeneous information. To examine the total utilization of general practice services, the total number of general practice contacts including all three groups of general practice contacts were extracted for the analysis. Physiotherapy contacts were included for the regression analysis without separating the way of contact since 95.4% were office visits. Additionally, all primary healthcare contacts including general practice contacts, physiotherapy contacts, and other outpatient contacts were summed up and extracted for the analyses.

### Participants’ characteristics and covariates

Participants’ characteristics were assessed during the clinical examinations between the years 2011 and 2013. Height and weight were measured, and BMI calculated as kilograms divided by the square of height in meters (kg/m^2^). Participants’ health status including current smoking status (yes/no) were inquired with questionnaires. Information on educational attainment was obtained from Statistics Finland in the year 2000 and classified into four groups: basic or less or unknown, upper secondary, lower tertiary including polytechnic, vocational, and bachelor’s degree, and upper tertiary referring to master’s degree or higher [21]. Charlson Comorbidity Index (CCI) [22] was calculated with the ICD-10 diagnostic codes [23] of the Care Register of Health Care, a national specialized healthcare register [24], since the codes were poorly reported in the Register of Primary Health Care Visits. CCI was available for 99.1% of the study participants.

### Statistical analyses

Participants’ characteristics are presented as means and standard deviations (SD) for continuous variables and as proportions for dichotomous or categorial variables. To assess possible differences in descriptive statistics between the frailty groups, the one-way ANOVA test, the Kruskall-Wallis test, and the Pearson chi square test were used when appropriate. We used the negative binomial regression model to investigate the possible association between frailty and each service use category because the data were overdispersed. The zero-truncated negative binomial model was used to model the association between frailty and the total number of primary healthcare contacts since there were no zeros. The individual exposure time was set to the models. The possible multicollinearity was tested with the variance inflation test. In total, four models were created. Model 1 is the crude model. Model 2 was adjusted for age and sex, Model 3 further for education and Model 4 additionally for BMI, CCI, and smoking status. The results are shown as incidence rate ratios (IRRs). Significance was set at p ˂ 0.05. Statistical analyses were performed with SPSS 26.0 for Windows (Version 26.0, 1989-2020, SPSS Inc., Armonk, NY, USA) and Stata 16 (Release 16, StataCorp LLC, College Station, TX).

## Results

### Characteristics of the study population

Table 1 displays the characteristics of the study population. Among all participants, 37 were classified as frail (3.5%), 427 pre-frail (40.1%), and 600 (56.4%) non-frail. As shown in Table 1, frail older adults scored higher in CCI (p < 0.001) and had more likely basic education or less (p < 0.001). They also had higher utilization rates of primary healthcare services (all p-values less than 0.001); especially the annual average utilization rate was two-fold compared to non-frail older adults (p < 0.001). A total of 61 (5.7%) participants died during the follow-up.

**Table 1:** Characteristics of the study population according to frailty classification

	All			Non-frail		Pre-frail		Frail		P^a^
	n	Mean (SD)		n	Mean (SD)	n	Mean (SD)	n	Mean (SD)	
**Participant charateristics assessed between the years 2011 and 2013**										
Age (years)	1064		71.0 (2.7)	600	70.7 (2.4)	427	71.4 (3.0)	37	71.4 (2.2)	0.001
Height (cm)	1052		168.4 (9.1)	595	168.9 (9.0)	421	168.0 (9.12)	36	165.4 (9.0)	0.037
Weight (kg)	1052		76.8 (14.3)	595	75.9 (13.2)	421	78.2 (15.4)	36	76.3 (17.0)	0.17
BMI (kg/m^2^)	1052		27.1 (4.5)	595	26.6 (4.0)	421	27.7 (4.9)	36	27.9 (6.2)	0.003
Women, %	595		55.9	332	55.3	238	55.7	25	67.6	0.35
Smoker, %	120		11.3	50	8.4	65	15.4	5	13.9	0.003
CCI	1054		3.5 (1.4)	592	3.4 (1.4)	425	3.7 (1.4)	37	4.3 (1.7)	<0.001
**Education**										<0.001
Basic or less or unknown, %	343		32.2	160	26.7	166	38.9	17	45.9	
Upper secondary, %	270		25.4	145	24.2	114	26.7	11	29.7	
Lower tertiary, %	296		27.8	193	32.2	97	22.7	6	16.2	
Upper tertiary, %	155		14.6	102	17.0	50	11.7	3	8.1	
**Primary healthcare utilization between the years 2013 and 2017**										
General practice office visits/year	1064		4.5 (5.1)	600	4.0 (5.1)	427	5.0 (4.8)	37	6.2 (5.9)	<0.001
General practice remote contacts/year	1064		3.9 (4.7)	600	3.3 (3.8)	427	4.8 (5.5)	37	5.1 (4.9)	<0.001
All general practice contacts/year	1064		10.3 (11.2)	600	8.9 (9.5)	427	11.8 (12.4)	37	15.2 (16.7)	<0.001
Physiotherapy contacts/year	1064		0.70 (2.2)	600	0.53 (1.9)	427	0.84 (2.3)	37	1.8 (3.2)	<0.001
All primary healthcare contacts	1064		12.0 (12.4)	600	10.2 (10.2)	427	13.8 (13.9)	37	19.8 (19.7)	<0.001

*SD *standard deviation, *BMI *body mass index, ^a^ statistical significance, *CCI* Charlson Comorbidity Index

### Primary healthcare utilization and frailty classification

Table 2 shows the results of the regression analyses. Compared to non-frailty, frailty was associated with a higher utilization rate of general practice office visits (fully adjusted IRR 1.31, 95% CI 1.01-1.69). The association was parallel among pre-frail older adults (fully adjusted IRR 1.21, 95% CI 1.10-1.34). There was no association between frailty and general practice remote contacts (Table 2). Pre-frailty, in turn, was associated with general practice remote contacts (fully adjusted IRR 1.39, 95% CI 1.22-1.57) and the total utilization of general practice contacts (fully adjusted IRR 1.25, 95% CI 1.12-1.40). Frailty was associated with the total utilization of general practice contacts in the first three regression models; the association, however, lost its statistical significance in the fully adjusted model.

Compared to non-frailty, frailty was strongly associated with more frequent physiotherapy contacts (fully adjusted IRR 2.97, 95% CI 1.49-5.91). Frailty was also associated with a higher total utilization rate of primary healthcare services (fully adjusted IRR 1.41, 95% CI 1.07-1.85) relative to non-frailty. The associations between pre-frailty and physiotherapy contacts or the total utilization rate of primary healthcare services were parallel to those of frailty but weaker (Table 2).

**Table 2:** Incidence rate ratios (IRRs) of primary healthcare utilization among non-frail, pre-frail and frail older adults

	Model 1^a^	Model 2^b^	Model 3^c^	Model 4^d^
	IRR (95% CI)	IRR (95% CI)	IRR (95% CI)	IRR (95% CI)
General practice office visits				
Non-frail	Ref.	Ref.	Ref.	Ref.
Pre-frail	1.28 (1.16, 1.41)^***^	1.25 (1.13, 1.37)^***^	1.21 (1.10, 1.33)^***^	1.21 (1.10, 1.34)^***^
Frail	1.44 (1.11, 1.87)^**^	1.41 (1.09, 1.83)^**^	1.33 (1.03, 1.72)^*^	1.31 (1.01, 1.69)^*^
General practice remote contacts				
Non-frail	Ref.	Ref.	Ref.	Ref.
Pre-frail	1.44 (1.27, 1.63)^***^	1.40 (1.24, 1.59)^***^	1.38 (1.22, 1.56)^***^	1.39 (1.22, 1.57)^***^
Frail	1.37 (0.98, 1.90)	1.31 (0.94, 1.82)	1.28 (0.92, 1.79)	1.18 (0.85, 1.65)
Total number of general practice contacts				
Non-frail	Ref.	Ref.	Ref.	Ref.
Pre-frail	1.33 (1.20, 1.49)^***^	1.30 (1.16, 1.45)^***^	1.26 (1.13, 1.41)^***^	1.25 (1.12, 1.40)^***^
Frail	1.46 (1.09,1.96)^*^	1.42 (1.06, 1.90)^*^	1.36 (1.02, 1.82)^*^	1.28 (0.95, 1.71)
Physiotherapy contacts				
Non-frail	Ref.	Ref.	Ref.	Ref.
Pre-frail	1.49 (1.13, 1.97)^**^	1.50 (1.14, 1.97)^**^	1.47 (1.12, 1.93)^**^	1.49 (1.14, 1.96)^**^
Frail	3.36 (1.63, 6.91)^**^	3.00 (1.47, 6.10)^**^	2.92 (1.45, 5.89)^**^	2.97 (1.49, 5.91)^**^
Total number of primary healthcare contacts				
Non-frail	Ref.	Ref.	Ref.	Ref.
Pre-frail	1.35 (1.22, 1.50)^***^	1.32 (1.19, 1.46)^***^	1.27 (1.15, 1.41)^***^	1.26 (1.13, 1.40)^***^
Frail	1.69 (1.28, 2.23)^***^	1.64 (1.25, 2.16)^***^	1.57 (1.20, 2.06)^**^	1.41 (1.07, 1.85)^*^

CI = Confidence interval

^a^ Crude model, *n* = 1064

^b^ Adjusted for frailty, age and sex, *n* = 1064

^c^ Adjusted for frailty, age, sex and education, *n* = 1064

^d^ Adjusted for frailty, age, sex, education, BMI, smoking and CCI, *n* = 1041

^*^ = *p* < 0.05, ^**^ = *p* < 0.01, ^***^ = *p* < 0.001

## Discussion

We found that frailty was associated with higher utilization rates of general practice office visits, physiotherapy contacts and the overall primary healthcare services compared to non-frail older adults. Moreover, we observed that pre-frailty was associated with general practice remote contacts and the total number of general practice contacts.

As shown in our study, besides general practice office visits, general practice remote contacts constitute a substantial proportion of general practice contacts among older adults. No studies, however, have examined the associations between frailty and general practice remote contacts or other visits excluding general practitioner visits. Apart from two studies [8, 12], previous studies have found frailty to be associated with general practitioner visits [7, 9–11, 13, 14] and general practice nurse visits [7]. We observed frailty to be associated with general practice office visits. Although our findings are not directly comparable since our data records consisted of both general practitioner and general practice nurse visits combined, the results can be considered parallel. Further, our results are similar compared to an English study which reported utilization rates of general practitioner or practice nurse visits to vary between 1.24 and 1.58 depending on the severity of frailty [7]. However, we observed no association between frailty and general practice remote contacts. These findings might indicate that frail older adults may prefer office visits over remote contacts, or that possible comorbidities and disability related to frailty warrant an office visit. However, the results should be interpreted with caution since the low prevalence of frailty in our study may challenge the detection of a possible association.

In contrast to frailty, we found that pre-frail older adults had a high utilization rate of general practice remote contacts. Additionally, as a new finding, we found a statistically significant association between pre-frailty and the total number of general practice contacts. However, although non-significant, frail older adults had a slightly higher utilization rate of general practice contacts. Therefore, further research is needed to establish the association since low prevalence of frailty in our study may hinder the detection of a possible association. Nevertheless, our results are similar to the findings of a Singaporean study which reported no association between frailty and general practitioner visits, but instead observed pre-frail older adults to use this service [8]. The authors noted, however, that their dataset consisted of governmental general practitioner visits excluding the private sector, which offers a substantial proportion of general practice services in Singapore. Therefore, they could not establish the association [8]. Our register data also excludes the private sector. In Finland, however, most older adults use public healthcare services independent of their household income levels [25]. Based on a national survey between the years 2013 and 2015, approximately 75% of the Finnish older adults had visited a general practitioner in a healthcare center [25]. In general, public healthcare is appreciated among Finnish older adults, and they wish care through public healthcare services if the need for care increases [26]. Therefore, the influence of possible unregistered contacts most likely would confound the results only to a small extent. Additionally, frailty was associated with the use of almost all specialized healthcare services in our previous study [27]. Thus, it might be possible that the health issues of frail older adults raise concern or require specialist opinion leading to referrals and treatments in specialized healthcare whereas pre-frail older adults utilize more general practice services, especially remote contacts. Therefore, the use of hospital-based services among frail older adults might decrease the utilization rates of general practice services. Further research, however, is needed to confirm these hypotheses.

The utilization rate of physiotherapy services was approximately three-fold higher among frail than non-frail older adults in our study. The results are in agreement with the two previous studies that found frailty to be associated with physiotherapy visits [11, 15]. The high utilization rate among frail older adults can be considered positive since exercise and resistance training interventions have shown signs of improving physical function in individuals with frailty [28, 29]. Little is known, however, about the effect of regular physiotherapy care on frailty or its cost-effectiveness. Physiotherapists, nevertheless, may play an important role in treating frailty through evidence-based rehabilitation, early recognition of frailty, safe use of assistive devices, and appropriate exercise prescriptions leading possibly to improvements in population health, reduced costs, and improvements in quality of life [30]. Thus, physiotherapy services may face challenges to adapt according to the needs of rapidly increasing number of frail older adults. Additionally, since almost all physiotherapy contacts in our study were office visits, it also refers to higher expenses compared to remote visits. However, we also observed a higher utilization rate of physiotherapy services among pre-frail compared to non-frail older adults, but the rate was approximately two times smaller compared to frail older adults. This finding emphasizes the prevention of frailty among pre-frail older adults in particular to maintain the sustainability and equal accessibility of physiotherapy services in the future.

Finally, we found an association between frailty and the total utilization of primary healthcare services. To the best of our knowledge, no previous studies have examined this. Our results suggest that high utilization rates of general practice office visits and physiotherapy contacts combined with smaller utilization rates of other outpatient primary healthcare services may contribute to the total service use among frail older adults.

Our findings also highlight the importance of public healthcare centers as early detectors of pre-frail and frail older adults. Improved education of healthcare personnel might serve as a channel to identify both groups. Based on our findings, physiotherapists in particular are likely to meet pre-frail and frail older adults while general practitioners and general practice nurses also play important role in the detection process. Thus, enhanced screening methods might be advantageous in primary healthcare settings to detect pre-frail and frail patients for further clinical evaluation. Once evaluated by educated health professional, comprehensive care plan could be implemented, and severe cases referred to a geriatrician [31]. Further, exercise interventions with or without nutritional supplements in primary healthcare settings may delay or reverse frailty [32, 33] and might be a feasible method to manage frailty in primary healthcare centers. In addition to these secondary prevention methods, primary prevention, like community education in local media and lectures on the importance of exercise given by health professionals [34], should also be considered to raise awareness of healthy ageing among older adults and their relatives. Together with enhanced screening methods, educated health professionals, exercise interventions, and exercise promotion campaigns it might be possible to decrease or delay the onset of pre-frailty and frailty and support healthy ageing among community-dwelling older adults. These steps towards age-friendly primary healthcare might ease the pressure of ageing populations on primary healthcare use as well.

Primary healthcare, offering a forefront of care for older adults in most countries and often with limited time and resources, needs to find effective ways to respond to the increasing demand of healthcare services. Our findings reveal important information on the role of frailty in primary healthcare service use and highlight frailty as a possible tool for designing and targeting primary healthcare services in the context of population ageing. Additionally, although we observed an association between frailty and the total utilization of primary healthcare services, pre-frailty was associated with the total utilization of general practice services, the backbone of primary healthcare. A high utilization rate of general practice remote contacts might have contributed to this. Although it is a cheaper way of contacting patients, more attention should be paid on this group, especially on the prevention of frailty to avoid further increases in healthcare use. In a broader context, appropriateness of the chosen level of care should be evaluated carefully since our studies may suggest that frail older adults utilize expensive specialized healthcare services in particular [27] whereas pre-frail older adults are frequent users of less expensive general practice services. These hypotheses, however, remain to be confirmed. Future studies are needed to examine the association between frailty and primary healthcare services, especially general practice remote contacts, and the total utilization of general practice and primary healthcare services.

Overall, our study provides evidence of the impact of frailty on the outpatient primary healthcare use in Finland, one of the fastest ageing countries in Europe with the current share of over-65-year-olds older adults being 22% [35]. Finland, among other high-income countries, needs to find ways to meet the needs of care of the growing number of older adults without losing sustainability and equality in primary healthcare. Knowledge about factors that increase outpatient primary healthcare use, such as frailty, is important to plan cost-effective treatments, targeted services, and preventative models to achieve this goal in the future.

The strengths of the study are the use of nationally registered data, which has previously been scarce in primary healthcare settings, and good-quality data from a unique birth cohort. The study also has limitations. In our study the prevalence of frailty was 3.5% which is slightly less than the lowest prevalence of 4.0% reported in a systematic review that examined the prevalence of frailty among community-dwelling older adults in high-income countries [36]. Individuals with poor health in particular might have declined to participate in the clinical examinations affecting the overall prevalence of frailty in our study. It may also hinder the detection of possible associations between frailty and healthcare use, especially affecting the results where no association was found. In that case our study might underestimate the healthcare use among frail older adults. Further, due to a large number of participants and several clinical measurements, the clinical examinations were performed between the years 2011 and 2013. This might have caused slight changes in frailty status among those few whose frailty assessment was conducted in the year 2011 compared to those who had the assessment in 2013. Additionally, the Register of Primary Health Care Visits lacks information on the private healthcare sector which might attract some of study participants leading to unregistered contacts. Further, we were unable to separate general practitioner and nurse visits from the dataset. Finally, the differences in the Finnish healthcare system might limit generalizability of the results to some countries.

In conclusion, we found that frailty predicted the overall use of primary healthcare services and most examined services. Pre-frailty, in turn, was associated with the utilization of general practice remote contacts and the total utilization of general practice services. These associations, however, remain to be confirmed. Primary healthcare needs to adapt outpatient services according to the needs of pre-frail and frail older adults and consider preventative interventions against frailty.

## Data Availability

The datasets generated and/or analyzed during the current study are available from the corresponding author on reasonable request.
